# Pre-pregnancy body mass index combined with peripheral blood PLGF, DCN, LDH, and UA in a risk prediction model for pre-eclampsia

**DOI:** 10.3389/fendo.2023.1297731

**Published:** 2024-01-08

**Authors:** Yanna Zhou, Chunhai Xiao, Yiting Yang

**Affiliations:** ^1^ Department of Obstetrics and Gynecology, Jinshan Branch of Shanghai Sixth People’s Hospital, Shanghai, China; ^2^ Department of Laboratory, Jinshan Branch of Shanghai Sixth People’s Hospital, Shanghai, China

**Keywords:** body mass index, preeclampsia, placental growth factor, blood placental growth factor, decorin, lactate dehydrogenase, uric acid

## Abstract

**Objective:**

This study analyzes the levels of peripheral blood placental growth factor (PLGF), body mass index (BMI), decorin (DCN), lactate dehydrogenase (LDH), uric acid (UA), and clinical indicators of patients with preeclampsia (PE), and establishes a predictive risk model of PE, which can provide a reference for early and effective prediction of PE.

**Methods:**

81 cases of pregnant women with PE who had regular prenatal checkups and delivered in Jinshan Branch of Shanghai Sixth People’s Hospital from June 2020 to December 2022 were analyzed, and 92 pregnant women with normal pregnancies who had their antenatal checkups and delivered at the hospital during the same period were selected as the control group. Clinical data and peripheral blood levels of PLGF, DCN, LDH, and UA were recorded, and the two groups were subjected to univariate screening and multifactorial logistic regression analysis. Based on the screening results, the diagnostic efficacy of PE was evaluated using the receiver operating characteristic (ROC) curve. Risk prediction nomogram model was constructed using *R* language. The Bootstrap method (self-sampling method) was used to validate and produce calibration plots; the decision curve analysis (DCA) was used to assess the clinical benefit rate of the model.

**Results:**

There were statistically significant differences in age, pre-pregnancy BMI, gestational weight gain, history of PE or family history, family history of hypertension, gestational diabetes mellitus, and history of renal disease between the two groups (P < 0.05). The results of multifactorial binary logistic stepwise regression revealed that peripheral blood levels of PLGF, DCN, LDH, UA, and pre-pregnancy BMI were independent influences on the occurrence of PE (P < 0.05). The area under the curve of PLGF, DCN, LDH, UA levels and pre-pregnancy BMI in the detection of PE was 0.952, with a sensitivity of 0.901 and a specificity of 0.913, which is better than a single clinical diagnostic indicator. The results of multifactor analysis were constructed as a nomogram model, and the mean absolute error of the calibration curve of the modeling set was 0.023, suggesting that the predictive probability of the model was generally compatible with the actual value. DCA showed the predictive model had a high net benefit in the range of 5% to 85%, suggesting that the model has clinical utility value.

**Conclusion:**

The occurrence of PE is related to the peripheral blood levels of PLGF, DCN, LDH, UA and pre-pregnancy BMI, and the combination of these indexes has a better clinical diagnostic value than a single index. The nomogram model constructed by using the above indicators can be used for the prediction of PE and has high predictive efficacy.

## 1 Introduction

Preeclampsia (PE) is the primary clinical manifestation of proteinuria, edema, and hypertension after 20 weeks of gestation. It is one of the more severe hypertensive disorders in pregnancy and has a serious adverse effect on the pregnancy outcome, quality of life, and physical and mental health of patients (1). Relevant epidemiologic studies have shown that the prevalence of PE ranges from 3% to 5% worldwide (2). Effective prevention and early treatment of PE in labor can prevent the life and health of mothers and infants from being threatened, whereas how to predict PE early in the clinic is of great significance. Vascular endothelial injury, placental “superficies”, and aseptic systemic inflammation are generally thought to be involved in the pathogenesis of PE, but the pathogenesis of PE has not yet been fully clarified (3). It has been pointed out that the pathogenesis of PE is very complex and comprehensive, and that there are two stages in the development of PE. The first stage is called placental dysplasia, and the second stage is insufficient maternal blood supply to the placental unit of the fetus (4). Some studies have found that maternal age and family history of hypertension are also associated with the development of PE, while others have suggested that changes in the concentration of placental growth factor (PLGF) are a key factor in the development of PE. PLGF is a cytokine secreted by the placenta, which can stimulate the proliferation and expansion of maternal blood vessels and participate in the regulation of blood pressure and circulatory system function in pregnant women. Studies have shown that PLGF levels are significantly lower in the blood of PE patients, and PLGF testing is important for early screening and treatment. Decorin (DCN) is a tiny proteoglycan containing leucine secreted by the stromal cells, which has an important role in the regulation of vasculogenesis in the organism (5). DCN can combine with vascular endothelial growth factor receptor-2 (VEGFR-2) on the extravillous trophoblast (EVT) to inhibit the proliferation of cells and antagonize hemangiogenesis, and thus some researchers have speculated that DCN may contribute to the pathological process of PE (6). It has been found that the level of DCN in the blood of PE patients is significantly elevated, and DCN can be used as one of the new predictive markers for PE, which is of great significance for understanding the pathogenic mechanism of PE and searching for new diagnostic and therapeutic targets. Lactate dehydrogenase (LDH) can promote the conversion of glucose metabolism into energy. Studies have shown that LDH levels in the blood of PE patients are often significantly elevated, which may be related to vascular endothelial cell damage, tissue hypoxia and so on. Therefore, LDH test can be used as one of the blood biochemical indexes for PE patients in the acute stage (7). Uric acid (UA) is an indicator that is routinely measured in metabolic diseases such as nephropathy. The kidney is one of the organs most likely to be involved in severe PE, and hyperuricemia is a common manifestation of PE, and the elevation of UA in pregnant women with PE usually precedes hypertension and proteinuria (8). This shows that the occurrence of PE is related to multiple factors, and it is difficult to accurately predict the risk of PE by a single indicator. Therefore, the four measurement factors of PLGF, DCN, LDH and UA have different importance in the prediction, diagnosis and assessment of PE, and should be comprehensively tested and analyzed according to the characteristics of the disease and clinical needs. Currently, early prediction and evaluation of PE are mainly performed by means of patient symptoms, risk factors, physical signs and auxiliary examinations (9, 10). Caterina et al. investigated a significant correlation between plasma miR-125b levels and PE, which together with maternal pre-pregnancy body mass index (BMI) provided a predictive model with an area under the curve of 0.85 (95% confidence interval, 0.70-1.00) (11). However, most of the previous studies used a single variable to predict PE and did not establish a prediction model with excellent sensitivity and specificity. Secondly, although there are some models for PE prediction in the clinic, they cannot be widely used in the clinic due to the complexity of operation or high cost. Therefore, it is still necessary to establish an accurate prediction model for PE that is multifactorial and clinically feasible. The aim of this study was to evaluate and validate the model using multifactorial binary logistic regression through serum PLGF, DCN, UA, LDH and clinical indicators, including the accuracy, sensitivity, specificity and receiver operating characteristic (ROC) curve of the model, to screen out the independent influences on the occurrence of PE, and to visualize the relationship between different factors and the risk of PE. Meanwhile, this study constructed a column-line diagram model to predict the risk of PE and explore the pathogenesis of PE.

## 2 Materials and methods

### 2.1 Collection of cases

This study analyzed 81 cases of pregnant women with PE who delivered at Jinshan Branch of Shanghai Sixth People’s Hospital with regular antenatal checkups from June 2020 to December 2022, and 92 cases of pregnant women who had normal pregnancies in our hospital during the same period were randomly selected as the control group after stratification by age and gestational week. Inclusion criteria: ① Cases met the relevant diagnosis of PE (12): systolic blood pressure ≥140 mmHg and/or diastolic blood pressure ≥90 mmHg after the 20th week of pregnancy, and any of the following: (a) urine protein ≥0.3 g/24 h; (b) random urine protein ≥(+); (c) no urine protein, but with the damages to important organs, such as the heart, liver, lungs, kidneys, or abnormal changes of the hematological system, gastroenterology, and nervous system, and placental-fetal involvement. ② Control group does not meet the diagnosis of PE; ③ single pregnancy, regular obstetric examination in our hospital; ④ natural pregnancy. Exclusion criteria: ① patients with primary hypertension; ② patients with severe mental illness who are unable to cooperate with the examination; ③ patients with severe infectious diseases; ④ patients with abnormal thyroid function; ⑤ patients with gestational diabetes mellitus, anemia, urinary tract infection; ⑥ patients with autoimmune diseases. ⑦ Those who use Cardioaspirin during pregnancy to prevent PE; The research was authorized by the Ethics Committee of Jinshan Branch of the Shanghai Sixth People’s Hospital (Grant No. jszxyy202233), and the patients were well informed and consented forms were signed.

### 2.2 Research methodologies

The baseline information of pregnant women in both groups was recorded, including age, gestational age, number of deliveries, whether they were primigravida, pre-pregnancy BMI, weight gain during pregnancy, history or family history of pre-eclampsia, family history of hypertension, gestational diabetes mellitus, and history of renal disease.

Collection of specimens: women with a diagnosis of preeclampsia in pregnancy who have not yet been treated with medication and women with a normal pregnancy, fasting for more than 8 hours in the morning, take elbow venous blood 5mL, 3500rpm, centrifuged for 5 min, take the supernatant, stored at -80°C for use.

Serum PLGF levels were measured using a Cobas e 602 electro-chemiluminescence immunoassay analyzer (Roche). LDH and UA levels were measured by Cobas c 501 automatic biochemical immunoassay analyzer (Roche).

Serum DCN levels were detected with an enzyme-linked immunosorbent assay (ELISA) by Wuhan Bost Biotechnology Co., Ltd. The tests were conducted in the same laboratory under the same person and strictly in compliance with the directions of the kits.

### 2.3 Monitoring indicators

Documenting Participants’ Clinical Data; Recording of PLGF, DCN, UA and LDH levels in peripheral blood; Multifactorial logistic regression was used to screen out the independence factors, and a nomogram model was constructed and validated based on the results.

### 2.4 Statistical analysis

Clinical data were presented as mean ± standard deviation ( $\bar{x}$ ± s), and t-test was applied for the comparison of the two groups; Statistical data were shown as frequency and rate, and chi-square test was used to compare the two groups; PE risk factors were analyzed by multifactorial logistic regression analysis, and P<0.05 was statistically significant. The diagnostic efficacy of PE was evaluated using the ROC curve. *R* language (*R* 3.4.3) software was used to visualize the results of the multifactorial analysis to obtain the nomogram model, and the computer simulation of the repeated sampling method (Bootstrap) was used to evaluate the calibration of the model; the clinical benefit rate of the model was evaluated using the decision curve analysis (DCA).

## 3 Results

### 3.1 Inclusion of the study population

**Figure 1:**
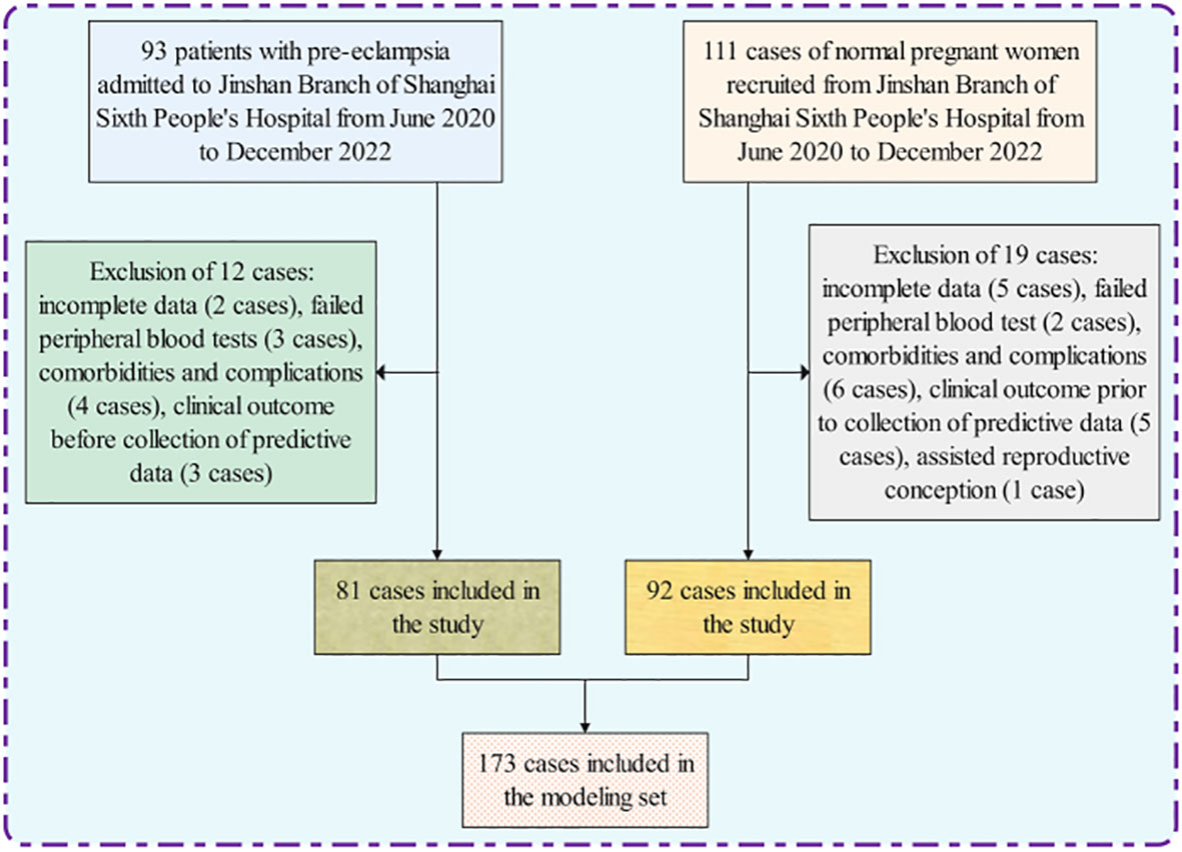
Flowchart for inclusion and grouping of modeled populations.

The population inclusion and grouping process of the study subjects are shown in **Figure 1**. In the study, 81 cases were in the PE group and 92 cases for the control group. There were 26 pregnant women with early-onset preeclampsia in the PE group, with a mean gestational week of 29.16 ± 0.82 (24-33.6/7 weeks). The abnormal intrauterine environment in patients with early-onset PE is prone to premature rupture of membranes, contractions and placental abruption, which can lead to preterm births. There is a close relationship between early-onset PE and preterm births, and the earlier the onset, the higher the risk of preterm births. In the control group, 29 pregnant women with the corresponding gestational week, with a mean gestational week of 29.32 ± 0.65 (24-33.6/7 weeks), showing no statistically significant difference (P>0.05); There were 55 pregnant women with late-onset preeclampsia in the PE group, with a mean gestational week of 36.23 ± 0.95 weeks (34-38.3/7 weeks), and 63 pregnant women in the control group with a mean gestational week of 36.53 ± 0.88 weeks (34-38.3/7 weeks), showing no statistically significant difference (P>0.05) (**Table 1**).

**Table 1:** Comparison of gestational weeks between pregnant women in the PE group (early and late) and the control group.

Groups	Cases	Mean gestational weeks	Groups	Cases	Mean gestational weeks
Early-onset PE	26	29.16 ± 0.82	Late-onset PE	55	36.23 ± 0.95
Control group	29	29.32 ± 0.65	Control group	63	36.53 ± 0.88
T		0.796	T		1.771
P		0.430	P		0.079

### 3.2 Comparison of general information and clinical indicators

As can be noted from **Table 2**, age, pre-pregnancy BMI, weight gain during pregnancy, history or family history of PE, family history of hypertension, gestational diabetes, history of renal disease, PLGF, DCN, UA and LDH levels were compared, and the differences were statistically significant. The probabilities corresponding to primigravida, cravidity and parity were 0.368, 0.435 and 0.234 respectively. Thus, they were not statistically significant.

**Table 2:** Comparison of clinical indicators in PE and control group ( $\bar{x}$ ± s).

Groups	pre-eclampsia (n=81)	control group (n=92)	*F/χ²*	T	P
Age (years)	30.84 ± 4.38	28.78 ± 4.39	/	-3.079	0.002
<26 years	14 (17.28%)	16 (17.39%)			
26 - <35 years	36 (44.45%)	41 (44.57%)			
≥ 5 years	31 (38.27%)	35 (38.04%)			
Primigravida [n (%)]	58 (71.60)	60 (65.22)	0.810	/	0.368
Cravidity	2.32 ± 0.74	2.41 ± 0.77	/	0.783	0.435
Parity	1.28 ± 0.48	1.37 ± 0.51	/	1.195	0.234
History or family history of PE [n (%)]	6 (7.41)	1 (1.09)	4.432	/	0.035
Family history of hypertension [n (%)]	22 (27.16)	13 (14.13)	4.532	/	0.033
Gestational diabetes [n (%)]	16 (19.75)	32 (16.30)	4.854	/	0.028
History of renal disease [n (%)]	4 (4.94)	0 (0.00)	4.651	/	0.031
Pre-pregnancy (kg/m²)	24.69 ± 1.32	22.63 ± 1.65	/	-9.113	<0.001
Weight gain during pregnancy (kg)	16.79± 4.61	14.93 ± 3.95	/	-2.830	0.005
PLGF (pg/mL)	216.68 ± 82.94	340.92 ± 94.55	/	9.207	<0.001
DCN (ng/ml)	0.73 ± 0.16	0.61 ± 0.28	/	-3.511	<0.001
LDH (U/L)	189.31 ± 22.89	168.41 ± 17.77	/	-6.642	<0.001
UA (μmol/L)	364.42 ± 65.42	317.99 ± 47.46	/	-5.280	<0.001

### 3.3 Multifactorial analysis of PE

The statistically significant indicators in **Table 2** were analyzed by univariate binary logistic regression, and the differences between the two groups in age, weight gain during pregnancy, pre-pregnancy BMI, history or family history of PE, family history of hypertension, diabetes mellitus in pregnancy, history of renal disease, PLGF, and DCN, UA, and LDH were statistically significant. The independent variables that were statistically significant within the above univariate analysis were incorporated into the regression equation. The results showed that PLGF, DCN, UA, LDH levels and pre-pregnancy BMI were independent influences on the development of PE (P < 0.05). The ORs were 0.986, 10.334, 1.013, 1.040, and 2.596, respectively, as shown in **Table 3**.

**Table 3:** Binary Logistic Regression Analysis of PE in Patients.

Indicators	β	S.E,	chi-square	df	P	OR	95% of OR C.I.	
							lower	upper
DCN	2.335	1.121	4.338	1	0.037	10.334	1.148	93.058
PLGF	-0.014	0.003	21.070	1	<0.001	0.986	0.980	0.992
LDH	0.040	0.013	9.105	1	0.003	1.040	1.014	1.068
UA	0.013	0.005	6.550	1	0.010	1.013	1.003	1.023
Pre-pregnancy BMI	0.954	0.194	24.109	1	<0.001	2.596	1.774	3.800
Constant	-31.893	5.940	28.829	1	<0.001	0.000	/	/

### 3.4 Predictive value of each index for PE

**Figure 2:**
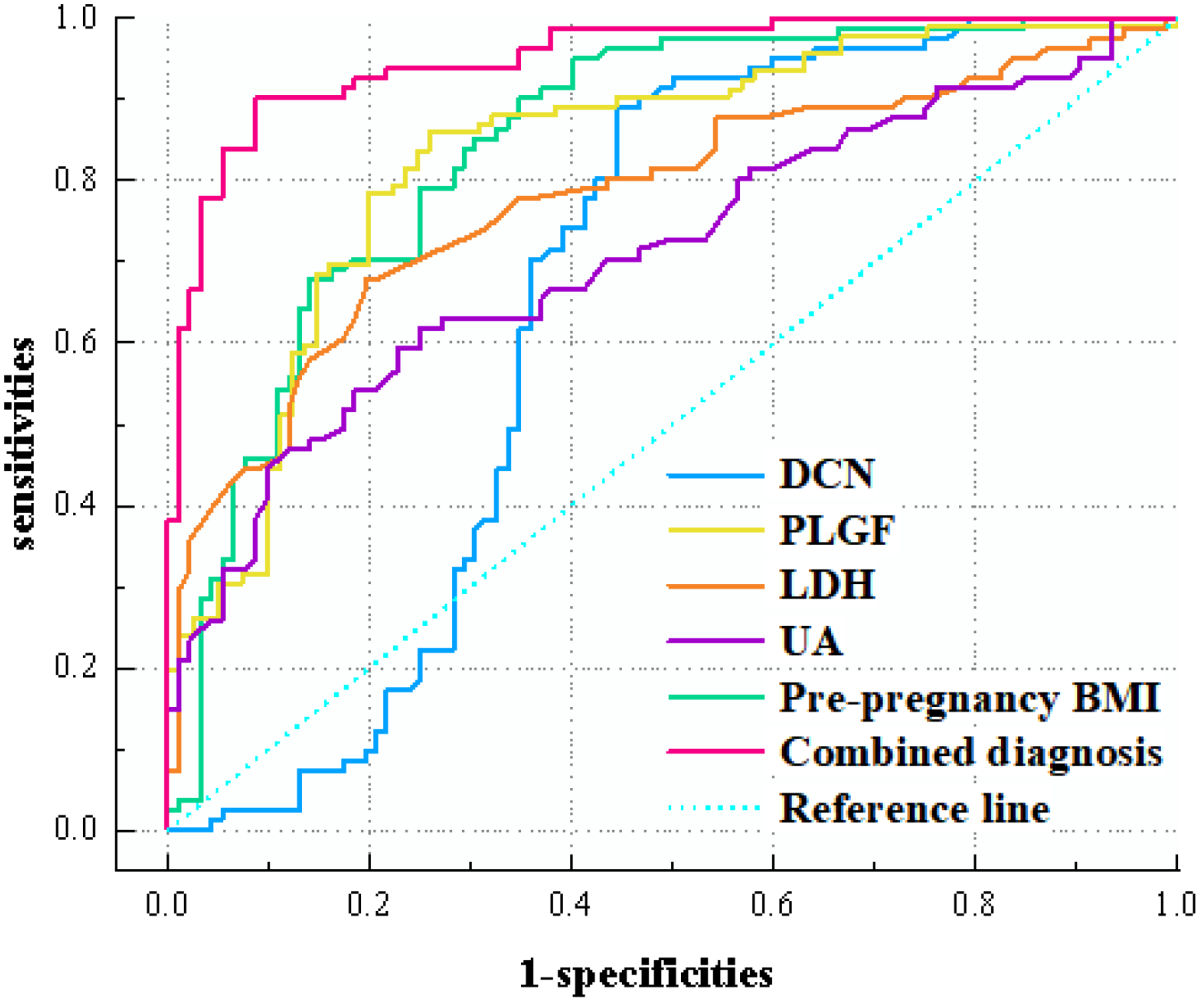
ROC curves of various indicators affecting the occurrence of PE in patients.

The ROC was used to determine the critical value of PLGF, DCN, UA, LDH levels, pre-pregnancy BMI, and the combined diagnostic value of each index for the prediction of PE (**Table 4** and **Figure 2**). The results showed that the area under the curve of the combined diagnostic curve of DCN, PLGF, LDH, UA, and pre-pregnancy BMI was 0.952, the sensitivity was 0.901, and the specificity was 0.913, which was the largest area under the curve of the combined diagnostic curve, and the sensitivity and specificity were higher than 90% compared to that of a single index, and it had a good clinical diagnostic value.

**Table 4:** ROC curve analysis by indicator.

	AUC	S.E.	P	95% of C.I.		threshold value	sensitivities	specificities	Youden index
				lower	upper				
DCN	0.655	0.044	<0.001	0.569	0.741	0.554	0.889	0.554	0.443
PLGF	0.833	0.031	<0.001	0.771	0.895	250.824	0.741	0.859	0.599
LDH	0.778	0.036	<0.001	0.708	0.849	182.500	0.679	0.804	0.483
UA	0.711	0.040	<0.001	0.633	0.789	348.500	0.617	0.750	0.367
Pre-pregnancy BMI	0.844	0.030	<0.001	0.785	0.902	23.144	0.901	0.652	0.553
Joint prediction probability	0.952	0.015	<0.001	0.922	0.981	0.514	0.901	0.913	0.814

### 3.5 Nomogram model for PE risk prediction

**Figure 3:**
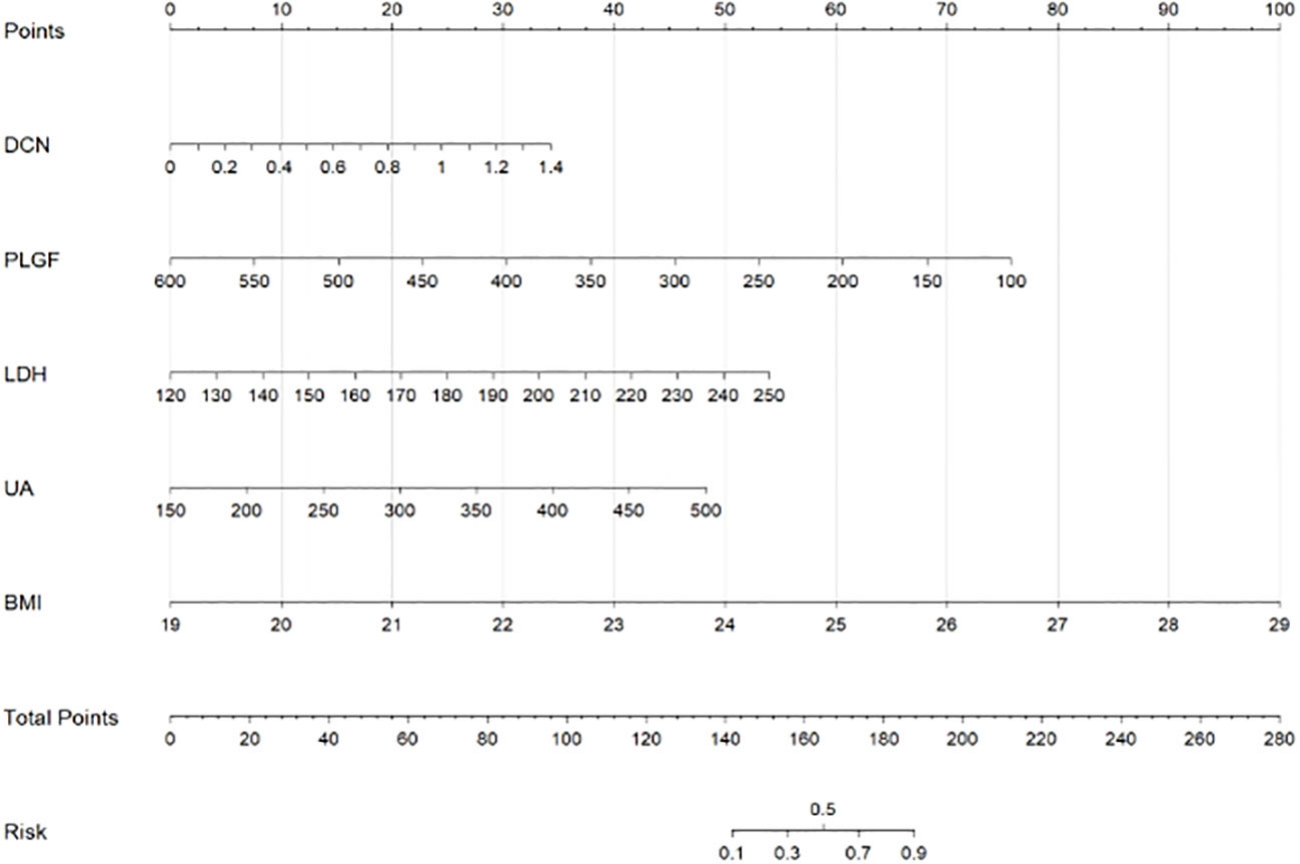
Scores on the nomogram for each of the independent influencing factors.

The independent factors screened by the multifactor logistic regression analysis were incorporated into the nomogram model. In the model, a score was obtained by positioning each predictor on the scale, and the overall score for each predictor was positioned on the total score axis, and corresponding risk coefficients reflect the patient’s risk of PE, as shown in **Figure 3**.

### 3.6 Model validation

**Figure 4:**
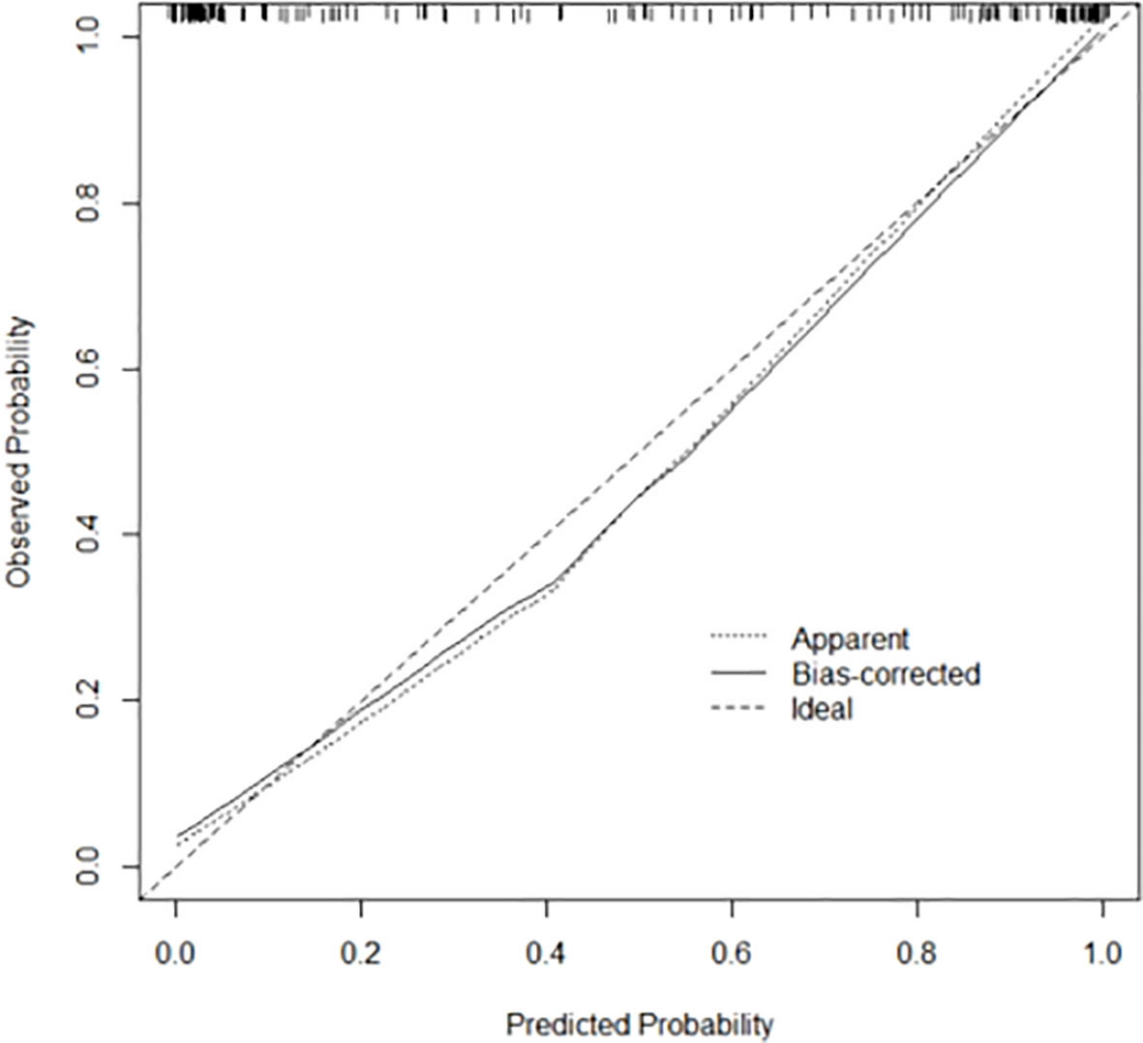
Calibration curve of Bootstrap method of prediction model.

Validation of the nomogram model by Bootstrap method with 1000 samples shows that the average absolute error of the calibration curve of the modeling set is 0.023, and the prediction curve is basically fitted to the standard curve, suggesting that the model prediction accuracy is high, as shown in **Figure 4**.

### 3.7 Decision curve

**Figure 5:**
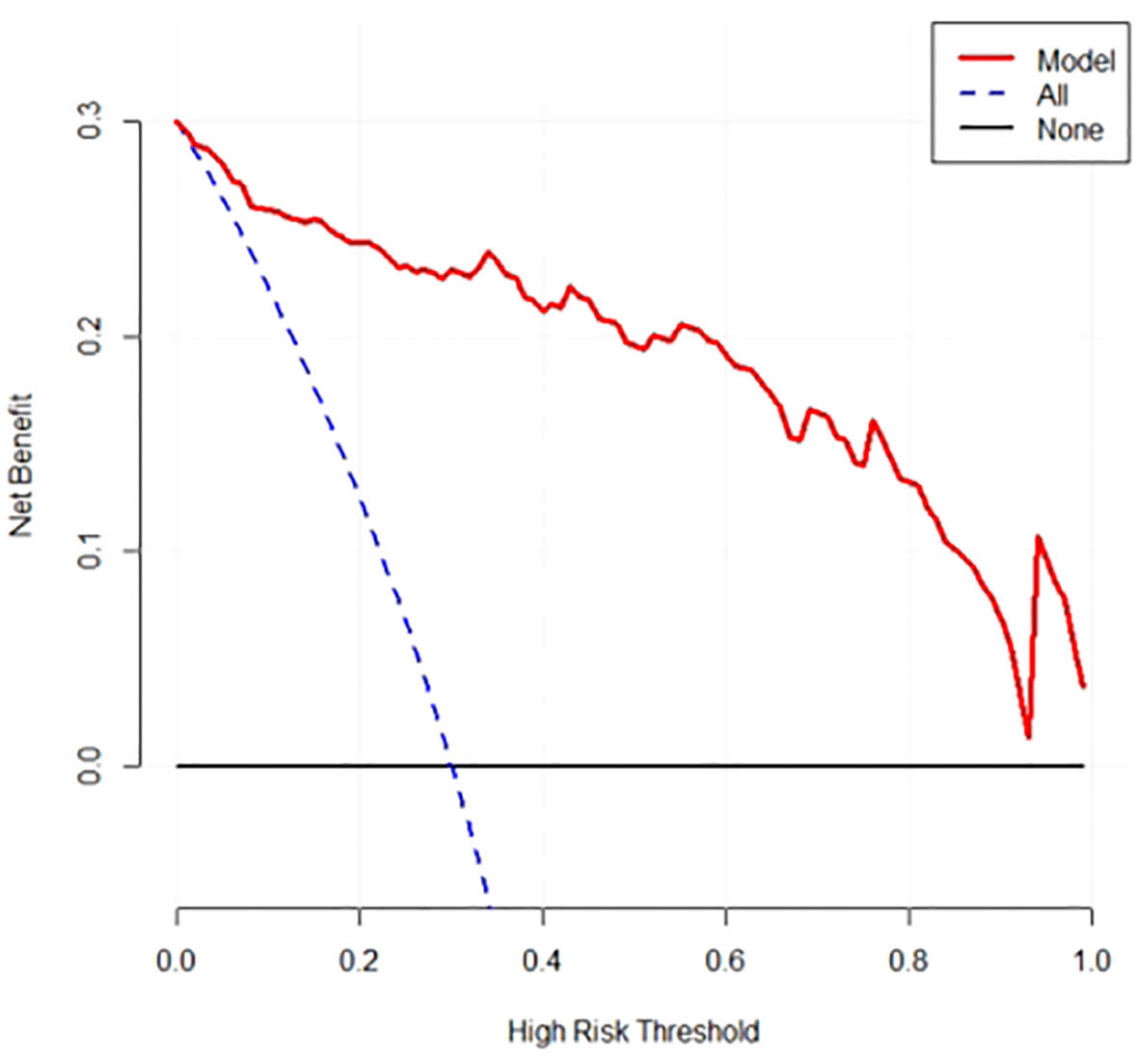
DCA Plot for Predictive Modeling.

DCA shows that the predictive model has high net benefits in the range of 5% to 85%, suggesting that in this model has clinical utility, see **Figure 5** for details.

## 4 Discussion

PE, as a pregnancy-specific complications of undetermined etiology, is also a complication of new-onset hypertension in pregnancy as well as a consequence that affects maternal organs and the foetal-placental systems. PE can cause placental abruption, eclampsia, hemolysis, elevated liver enzymes and low platelets (HELLP) syndrome due to its rapid onset, thus posing a serious threat to the welfare of the mother and child. Recent research has indicated that PE also contributes to maternal and pediatric ill health in later life (13). Therefore, how to predict and prevent PE at an early stage is currently a widespread concern in both clinical and research fields. Currently, there is a wide variety of factors that contribute to PE, such as nutritional deficiencies, placental ischemia and hypoxia, inflammatory immune hyperactivation, insulin resistance, and damage to the vascular endothelium, to name a few. PE is a disorder of placental origin, and trophoblast cells are a major component of the placenta. Many studies have suggested that insufficient infiltration of trophoblast cells results in remodeling of spiral arteries and reduced placental vasculature, which allows an imbalance in immune function, leading to chronic inflammation (14). Therefore, the effect of inflammatory response in the pathogenesis and progressive of PE has received extensive attention from scholars. The successful pregnancy needs the maternal immune system to be sufficiently tolerant to the foetus, but it has been observed that there is an over-activation of the immune response and inflammation in the organism of patients with PE, both systemically and locally (15). This study collected demographic, clinical, and laboratory data related to PE, including age, history of hypertensive disorders, and numerous measurements in the blood, and used logistic regression univariate and multivariate analyses to screen for BMI during pregnancy and serum PLGF, DCN, LDH, and UA as the largest independent influences related to PE. This combination effect is broad and scientific both in terms of the selection of the range of indicators and the judgmental selection of key factors. Therefore, the results of this study are important guidance and reference for the early and accurate prediction of PE.

### 4.1 Comparison of clinical data between PE patients and control pregnant women

The findings of the study further revealed that the difference in age, history or family history of PE, family history of hypertension, gestational comorbidity with diabetes mellitus, and history of renal disorders was statistically significant (P < 0.05) when compared between the two groups. Sandström et al. (16) showed that age is an independent factor in the development of PE. It has been reported in the literature (17) that the risk of PE in pregnant women aged more than 35 years is two to four times higher than that in those aged less than 35 years, which may be related to the higher basal blood pressure in older pregnant women; moreover, the aging of uterine blood vessels with the increase of maternal age is also an important reason for the increased risk of PE. A retrospective cohort study by Bijl et al. (18) included women with a medical history of PE and developed a prediction model for recurrent PE, with the final predictors including maternal age and genetic factors such as the presence of cardiovascular disease and placenta-derived disease in first-degree relatives. One study (19) found that the risk coefficients for PE in pregnant women with paternal and maternal hypertension were 3.27 and 3.83, respectively, and the risk coefficients were as high as 10.21 if both parents were hypertensive. Kivioja et al. (20) compared 1514 pregnant women with PE with 983 control pregnant women in the Finnish Genetics of Preeclampsia Consortium cohort study (FINNPEC study) and showed that a high polygenic risk score for hypertension (>95th percentile) was associated with an increased risk of recurrent PE. The results of this study showed that a history or family history of PE and a family history of hypertension were influential factors for PE, suggesting that the occurrence of PE is genetically related. Tabet et al. (21) indicated that gestational diabetes mellitus and chronic hypotension are major contributing risk elements for the recurrence of PE. Another study (22) pointed out that high body mass index and high fasting glucose, are risk factors for gestational diabetes mellitus complicating PE (OR value >1, P<0.05). In a study by Hussein & Lafayette (23), it was found that glomerular epithelial cell damage and alterations in the renin angiotensin aldosterone system in pregnant women with comorbid chronic nephritis can lead to the development of PE.

The present study showed that the difference between pre-pregnancy BMI and weight increase during pregnancy was statistically significant and that pre-pregnancy BMI was an independently risk factor in the development of eclampsia for patients. Excessive pre-pregnancy BMI has a significant positive correlation with the occurrence of PE. Therefore, the importance of pre-pregnancy BMI in PE risk prediction model should not be ignored, effectively guiding women to control their pre-pregnancy body weight and reduce the risk of PE. Liu et al. showed a significantly higher incidence of PE in overweight or obese (BMI ≥ 25 kg/m^2^) pregnant women (24). Pre-pregnancy BMI is an important predictor of gestational hypertension (GH) and PE. Pre-pregnancy obviously obesity and overweight are considered most potential to increase the chances of having GH and PE as compared to other risk factors. A rapid gestational weight gain (GWG) is also a significant predicator, which works in conjunction with an excessive BMI (25). An observational study conducted by Robillard et al. (26), which included 1,736 pregnant women with PE in singleton pregnancies from 2001-2018, investigated the effect of pre-pregnancy BMI on early-onset PE and late-onset PE, and the results suggested that pre-pregnancy BMI was more closely associated with late-onset PE. Tabet et al. (21) demonstrated that inter-pregnancy weight gain was associated with an increased risk of recurrent PE in a “dose-response” manner, and this risk was more pronounced in first-time pregnancies with low or normal weight.

### 4.2 Comparison of clinical indicators between pregnant women with PE and control group

Serologic indicators are one of the most important methods to predict PE. This study reveals that multifactorial binary logistic stepwise regression **shows** that peripheral blood levels of PLGF, DCN, UA, and LDH are independent factors influencing the development of PE in the patients (P < 0.05). In PE patients, PLGF levels are significantly lower, DCN, LDH and UA levels are often significantly higher. These abnormalities reflect a series of physiological, pathological, and metabolic changes in the mother’s body, so their role in the risk prediction model of PE can help to better understand, predict, and intervene in the occurrence and development of PE.

The results of the present study are consistent with the above conclusions that PLGF levels in patients with PE were significantly less than the control group, and the results of logistic regression showed that PLGF levels were an independent factor affecting the occurrence of eclampsia in patients. The possible reason for this is that lower PLGF expression reduces the proliferation and chemotaxis of placental trophoblasts and vascular endothelial cells, which in turn inhibits the process of placental and fetal angiogenesis, and placental vascular remodeling is impaired, leading to the development of PE. Placental angiogenesis requires complex interactions between vascular growth factors and their receptors. Angiogenic factors have been shown to be key components in regulating the activity of trophoblasts and allowing them to play a functional role, and an imbalance in their regulation initiates a pathological mechanism for the development of PE, leading to insufficient invasion of trophoblasts, and hypoxia in uterine and placental tissues. PLGF is an important member of the vascular endothelial growth factor (VEGF) family in the organism, and it is a glycoprotein homodimer molecule with physiological functions of activating vascular endothelial cells and promoting cell migration and proliferation. Angiogenic factors, such as PLGF, has shown promise in the prediction of this disease (27). Kosinska-Kaczynska et al.’s study (28) noted that PLGF levels in serum and placenta of pregnant women with PE were significantly lower than those of normal pregnant women. The National Institute for Health and Care Excellence had endorsed the use of PLGF as an adjunct to clinical assessment to rule out PE (29).

In this study, peripheral blood DCN levels are elevated for patients with PE relative to healthy controls. It is inferred that DCN participates in the intra-organizational immune-inflammatory response in patients with PE, and plays an important role in its pathogenesis. DCN are small leucine-rich proteoglycans composed of endometrial cells, chorionic villous mesenchymal stromal cells, chondrocytes, and dermal fibroblasts, which play an important role in regulating angiogenesis, placental trophoblast invasion, autophagy, and other cellular functions. Iacob et al. (30) have previously stated that aberrant release of certain metaphase-derived DCN propeptides or intramembranous DCN overexpression may play an important influence in the etiopathogenesis of patients with PE, and thus DCN is able to delay vascular response or angiogenesis by competitively binding to VEGFR-2 and blocking other peptides that activate endothelial activity. Furthermore, DCN also mediates a dual disorder of extravillous trophoblast angiogenesis and endovascular differentiation (31). The inhibition or promotion of DCN on human vasculature depends on the angiogenic microenvironment, and DCN actively promotes angiogenesis during normal organ development and within the vascular microenvironment (32), but has the opposite effect in the state of pregnancy or in tumors (33). Liu et al. (34) has showed that DCN regulates the aerobic metabolism and anaerobic glycolysis of trophoblast cells, induces autophagy and apoptosis of HTR-8 cells through inhibition of the c-Met/Akt/mTOR pathway, and regulates the occurrence and development of PE. DCN, as a negative regulator, binds to VEGFR-2 and then suppresses the proliferation, relocation, and intrusion of EVT cells and facilitates apoptosis, thereby influencing the process of the maternal utero-placental vascular bed development and spiral artery remodeling, leading to vascular endothelial damage (35).

Consistent with previous research, serum UA level was an independent influence on PE in the patients of the present study. Blood uric acid levels change dynamically during pregnancy, and elevated serum uric acid is a common clinical symptom in pregnant women with PE, hypertension and diabetes (36). Increased serum uric acid concentration increases the risk of hypertension and PE during pregnancy (37). Study (38) found that a large number of free radicals are produced in patients with PE, leading to increased UA synthesis. High UA can affect trophoblast invasion and interfere with the physiologic recasting of small placental spiral arteries, leading to the development of PE (39).

This study also analyzed the correlation between LDH and PE. LDH is one of the important oxidoreductases in organisms, which plays a very important role in redox, detoxification, and certain physiological activities in organisms. The level of LDH is related to the degree of damage to endothelial cells in hypertensive disorders of pregnancy, and the content of intracellular LDH increases accordingly with the prolongation of ischemia and hypoxia time. It has also been shown that LDH can be used as a biomarker for the diagnosis of PE and an effective marker for prognostication (40). Moreover, multiorgan dysfunction caused by vascular endothelial injury in PE can lead to a large leakage of LDH, and cellular dysfunction leads to increased serum LDH levels (41). Some scholars (42) found that the sensitivity of diagnosing PE was 0.838 and the specificity was 0.714, when LDH was at the critical level of 250 U/L.

Several models have been developed to predict the risk of developing PE in early pregnancy with variable sensitivity, and the risk of developing PE is better identified by combining clinical risk factors, biochemical and ultrasound indicators than by a single indicator (43–46). In this study, serum PLGF, DCN, LDH, UA levels and pre-pregnancy BMI had a combined diagnostic curve area under the curve of 0.952 for preeclampsia, with a sensitivity of 0.901 and a specificity of 0.913, which had a good clinical diagnostic value compared with a single indicator. The results of multifactor analysis constructed a column-line graph model, suggesting that the predictive probability of the model is basically consistent with the actual value, suggesting that the model has clinical practical value.

Advantages of multi-indicator joint prediction: The establishment and optimization of a multi-indicator joint prediction model based on pre-pregnancy BMI and peripheral blood indicators has obvious advantages in terms of accuracy and sensitivity in the prediction of the risk of PE. Because there may be synergistic or inhibitory effects between different indicators, the establishment of a multi-indicator joint prediction model can help to comprehensively analyze the trend and intensity of changes, reduce the impact of misjudgment of individual indicators, and improve the prediction accuracy of the model.

## 5 Conclusions

In summary, for pregnant women with high pre-pregnancy BMI, further monitoring of serum levels of PLGF, DCN, LDH and UA, and comprehensive evaluation of the results of monitoring during pregnancy, can be used to predict the risk of PE in a scientifically sound manner. The accuracy and sensitivity of risk prediction can be further improved, which can help clinicians to identify the high-risk groups of PE in time and take effective preventive and intervention measures, and also play an important role in the diagnosis and treatment of PE, preventing complications and safeguarding the health of the pregnant women and the fetus. Therefore, the model can be applied in clinical practice as a powerful tool for the prevention and control of PE. However, there is some heterogeneity across studies in terms of sampling populations, study methods, study quality and measurement outcomes. Further studies with multicenter and large sample sizes are needed.

## Data availability statement

The raw data supporting the conclusions of this article will be made available by the authors, without undue reservation.

## Ethics statement

Written informed consent was obtained from the individual(s) for the publication of any potentially identifiable images or data included in this article.

## Author contributions

YZ: Conceptualization, Data curation, Formal analysis, Funding acquisition, Investigation, Methodology, Project administration, Resources, Validation, Writing – original draft, Writing – review & editing. CX: Conceptualization, Data curation, Resources, Supervision, Validation, Writing – review & editing. YY: Data curation, Project administration, Software, Validation, Visualization, Writing – original draft.
